# Early Embryonic Expression of *AP-2α* Is Critical for Cardiovascular Development

**DOI:** 10.3390/jcdd7030027

**Published:** 2020-07-23

**Authors:** Amy-Leigh Johnson, Jürgen E. Schneider, Timothy J. Mohun, Trevor Williams, Shoumo Bhattacharya, Deborah J. Henderson, Helen M. Phillips, Simon D. Bamforth

**Affiliations:** 1 Newcastle University Biosciences Institute, Centre for Life, Newcastle NE1 3BZ, UK; bs06alj@gmail.com (A.-L.J.); deborah.henderson@newcastle.ac.uk (D.J.H.); helen.phillips@newcastle.ac.uk (H.M.P.); 2 Biomedical Imaging, University of Leeds, Leeds LS2 9JT, UK; J.E.Schneider@leeds.ac.uk; 3 The Francis Crick Institute, London NW1 1AT, UK; tim.mohun@gmail.com; 4 Department of Craniofacial Biology, University of Colorado Anshutz Medical Campus, Aurora, CO 80045, USA; trevor.williams@cuanschutz.edu; 5 Department of Cardiovascular Medicine, University of Oxford, Wellcome Trust Centre for Human Genetics, Oxford OX3 7BN, UK; sbhattac@well.ox.ac.uk

**Keywords:** transcription factor AP-2α, cardiovascular development, outflow tract, pharyngeal arch artery, neural crest cell, pharyngeal ectoderm

## Abstract

Congenital cardiovascular malformation is a common birth defect incorporating abnormalities of the outflow tract and aortic arch arteries, and mice deficient in the transcription factor *AP-2α* (*Tcfap2a*) present with complex defects affecting these structures. AP-2α is expressed in the pharyngeal surface ectoderm and neural crest at mid-embryogenesis in the mouse, but the precise tissue compartment in which *AP-2α* is required for cardiovascular development has not been identified. In this study we describe the fully penetrant *AP-2α* deficient cardiovascular phenotype on a C57Bl/6J genetic background and show that this is associated with increased apoptosis in the pharyngeal ectoderm. Neural crest cell migration into the pharyngeal arches was not affected. Cre-expressing transgenic mice were used in conjunction with an *AP-2α* conditional allele to examine the effect of deleting *AP-2α* from the pharyngeal surface ectoderm and the neural crest, either individually or in combination, as well as the second heart field. This, surprisingly, was unable to fully recapitulate the global *AP-2α* deficient cardiovascular phenotype. The outflow tract and arch artery phenotype was, however, recapitulated through early embryonic Cre-mediated recombination. These findings indicate that *AP-2α* has a complex influence on cardiovascular development either being required very early in embryogenesis and/or having a redundant function in many tissue layers.

## 1. Introduction

Congenital cardiovascular malformation is a major cause of morbidity and death in childhood, with an incidence of 1% in live-born infants [1,2]. A significant proportion of these malformations affect the outflow tract (OFT) and aortic arch arteries, resulting in the mixing of oxygenated and deoxygenated blood and the inefficient systemic delivery of blood to the body. The mammalian OFT develops to separate the common trunk exiting the heart into the aorta and pulmonary trunk, and this process begins at embryonic day (E) 10.5 in the mouse. Cardiac jelly in the OFT is invaded by neural crest cells (NCC), forming the two major OFT cushions which spiral around each other with one positioned septally and caudally, and the other parietally and cranially [3]. By E11.5 the aortopulmonary septum, which is comprised of second heart field (SHF) cells and NCC [4], protrudes into the OFT cavity and separates the future aorta and pulmonary trunk vessels by fusing with the distal ends of the OFT cushions. The proximal intra-pericardial components of the aorta and the pulmonary trunk are separated from the ventricular outflow tracts by the aortopulmonary septal complex, comprising of neural crest-derived mesenchyme. After E12.5 the major and intercalated OFT cushions remodel to form the tricuspid arterial valves and subsequently the aortic root is transferred to the left ventricle, with the pulmonary trunk remaining as the sole outlet from the right ventricle [3].

The great arteries of the mammalian heart are derived from the pharyngeal arch arteries (PAAs) which form within the pharyngeal arches. These are temporary embryological structures that develop as a series of repeated protuberances on either side of the developing pharynx [5]. The pharyngeal arches consist of paraxial mesoderm- and NCC-derived mesenchyme, enclosed by two epithelial layers—the pharyngeal ectoderm and endoderm. The PAAs form symmetrically and sequentially during early embryogenesis in a cranial to caudal sequence and are rapidly remodelled to form the asymmetric aortic arch arteries seen in the adult [6,7]. The first two pairs of PAAs degenerate early and contribute to the vascular plexus within the forming jaw, and the third PAAs, on both the right and the left, form the common carotid arteries. The fourth PAA persists on the right as the proximal region of the subclavian artery, whilst on the left it contributes to the section of the mature aortic arch between the origins of the left common carotid artery and the left subclavian artery. The right sixth PAA regresses, while the left sixth PAA forms the arterial duct. The development of the PAAs appears to be particularly sensitive to insult during embryonic life, and a variety of teratogens, (e.g., retinoic acid [8,9]), blood flow perturbations [10,11] and gene mutations in mouse models [12] result in defects of the cardiovascular system. Additionally, neural crest ablation in the mouse gives rise to a wide spectrum of OFT and aortic arch patterning defects [13,14]. Moreover, gene interactions within the pharyngeal epithelia are crucial for PAA development [15,16,17].

Mice lacking the non-DNA binding transcription factor, *Cited2*, die in utero with complex cardiovascular and neural tube malformations [18,19,20,21] and CITED2 has been shown to physically interact with, and co-activate, AP-2α (*TFAP2A* in humans) [20]. *AP-2α*, similar to *Cited2*, is necessary for cardiovascular and neural tube development [22,23,24]. Mutations in *TFAP2A* have been identified in patients with Peters’ anomaly [25], which frequently has comorbidities with congenital heart defects [26,27], and cause branchio-oculo-facial syndrome, a disease characterised by a branchial (or cutaneous) defect, abnormalities of the eyes and a distinct facial phenotype [28]. These patients may also present with cardiovascular defects, such as atrial septal defect and Tetralogy of Fallot [29,30]. Additionally, *TFAP2A* has been associated with the negative regulation of the T-box gene, *TBX20*, the expression of which is increased in atrial and ventricular biopsies of patients with Tetralogy of Fallot [31]. Other AP-2 family members have been implicated in congenital heart defects, with mutations in *TFAP2B*, causing Char syndrome, where patients present with persistent arterial duct [32], and decreased TFAP2C levels have been identified in cardiac biopsies of Tetralogy of Fallot patients [31].

In the mouse, AP-2α is expressed in the surface ectoderm and lateral edge of the neural plate at the 3-somite stage (~E8.0) [33], and in the neural crest cells migrating from the hindbrain region at the 5-somite stage [34]. By E9.5, AP-2α is expressed in the neural crest derived mesenchyme of the pharyngeal arches, and the pharyngeal ectoderm, and this expression extends into the mesenchyme and ectoderm of pharyngeal arches four and six by E10.5 [24]. The cardiovascular defects observed in *AP-2α*-null mice affect the OFT (e.g., common arterial trunk) and the aortic arch arteries (e.g., interrupted aortic arch) but these present differently depending on the genetic background used [22,23,24]. Although the OFT and arch artery defects are characteristic of a NCC phenotype [35], published genetic evidence using *Cre*-lox techniques suggests that NCC deletion of *AP-2α* does not result in cardiovascular malformations although there is perinatal lethality associated with neural tube defects and cleft palate [36]. This raises important questions as to how cardiovascular defects arise that appear to be NCC-related, but which have been shown not to be caused by a cell-autonomous NCC phenotype.

In this study we describe the fully penetrant *AP-2α*-null cardiovascular phenotype on an enriched C57Bl/6J genetic background. We also sought to identify the tissue-specific requirement of *AP-2α* in cardiovascular development using null and conditional alleles of *AP-2α* and *Cre* recombinase expressing transgenic mice. We demonstrate that an early embryonic deletion of *AP-2α* is required to recapitulate the cardiovascular defects seen in the constitutive knockout.

## 2. Materials and Methods

### 2.1. Mice

All mice used in this study have been described elsewhere: *Tcfap2a^lacZ-KI^* (referred to as *AP-2α*^+/−^) [24], a floxed allele of *Tcfap2a* (referred to as *AP-2α^flox^*) [36]; *Foxg1Cre* [37]; *Nkx2-5Cre* [38]; *Wnt1Cre* [39]; *Isl1Cre* [40]; *PGKCre* [41]; *R26R^lacZ^* [42]; *R26R^eyfp^* [43]. All mice used in this study were backcrossed using a traditional congenic breeding strategy [44] for sufficient generations onto C57Bl/6J to give an average contribution of >90% C57Bl/6J genomic DNA, and were genotyped by standard PCR using allele-specific primers (sequences available on request). All studies involving animals were performed in accordance with the UK Home Office Animals (Scientific Procedures) Act 1986.

### 2.2. Breeding

Stud male mice were produced by crossing *AP-2α*^+/−^ with each *Cre* strain. The *AP-2α^flox^* mice were crossed with *R26R^eyfp^* to produce double homozygous females. Male and female mice were mated and the detection of a vaginal plug the next morning was considered to be embryonic day (E) 0.5. Pregnant females were culled on the required day and embryos collected. Embryos at E9.5–E11.5 were staged by somite counting.

### 2.3. Imaging

The magnetic resonance imaging (MRI) of E15.5 embryos was performed on a 9.4T MR system and analysed and segmented as described [45]. Following MRI, selected embryos were processed for standard histology and haematoxylin and eosin staining. Mid-gestation embryos (E10.5 and E11.5) were analysed by High Resolution Episcopic Microscopy (HREM) as described [46,47]. To create the 3-dimensional (3D) reconstructions of the heart and arch arteries, MRI and HREM images were converted into volume datasets and segmented using Amira software (Thermo Fisher Scientific, Waltham, MA, USA). Structures were manually outlined using the label field function of Amira and surface rendered to produce the 3D images [45]. X-Gal staining to reveal *lacZ* activity was performed using standard procedures. To visualise the patency of pharyngeal arch arteries at E10.5, embryos were injected with India ink via the left ventricle with pulled Pasteur pipettes.

### 2.4. Immunohistochemistry

Embryos were embedded in paraffin wax and sectioned. Slides were dewaxed, rehydrated and immunostained with the following primary antibodies: mouse anti-AP-2α (3B5, Santa Cruz Biotechnology, Dallas, TX, USA; Catalogue number: sc-12726); rat anti-mouse CD-31 (BD Biosciences, San Jose, CA, USA; Catalogue number: 550274); chicken anti-GFP (Abcam, Cambridge, UK; Catalogue number: ab13970); anti-cleaved caspase3 (Cell Signalling Technology, Danvers, MA, USA; Catalogue number: 9661); anti-phospho-histone H3 (Millipore, Watford, UK; Catalogue number: 06-570). For cell fate analysis, three embryos for each genotype (*n* ≥ 3 sections per embryo) were assessed and the number of positively stained cells divided by the total number of cells to give an apoptotic or proliferative index. NCC were labelled genetically with eYFP using the *Wnt1Cre* allele, and sections immunostained with the anti-GFP antibody, and the NCC counted (*n* = 3 embryos per genotype; *n* ≥ 3 sections per embryo). Cell counting was performed using ImageJ software (National Institutes of Health, Bethesda, MD, USA) using the cell counter feature.

### 2.5. RT-PCR

Embryos were collected at E9.5 and the pharyngeal arch region dissected free in ice-cold PBS. Total RNA was extracted using TRIzol and a PureLink™ RNA mini spin column kit, and cDNA synthesis was carried out using the High Capacity cDNA reverse transcription kit (all from ThermoFisher Scientific, Waltham, MA, USA). Oligonucleotides were designed to identify the *AP-2α* wild-type (5′-TTAAGAAAGGCCCCGTGTCCCTG and 5′-CGTTGGGGTTTACCACGCCAC), floxed and recombined alleles (5′-TTAAGAAAGGCCCCGTGTCCCTG and 5′-TAACCGCTGCACACACCGCC), and *Gapdh* (5′-TGTGCAGTGCCAGCCTCGTC and 5′-TGACCAGGCGCCCAATACGG).

### 2.6. Western Blotting

Embryos were collected at E10.5, dissected in ice-cold PBS and lysed in 300 μL Laemmli buffer. Lysates were electrophoresed through a 10% polyacrylamide gel and subsequently transferred to PVDF membrane. The membrane was blocked in 5% milk solution and incubated with AP-2α antibody, diluted 1:1000. Secondary antibody was a polyclonal goat anti-mouse HRP (Agilent, Santa Clara, CA, USA) diluted 1:10,000.

### 2.7. Statistical Analysis

Cell counts were analysed using a two-tailed unpaired *t*-test (Prism 8.01 software, GraphPad, San Diego, CA USA). Fisher’s exact test was used to compare defect frequencies between different genotypes (SPSS). Groups were considered significantly different when *p* < 0.05.

## 3. Results

### 3.1. Cardiovascular Phenotype in C57Bl/6J AP-2α^−/−^ Embryos

**Figure 1:**
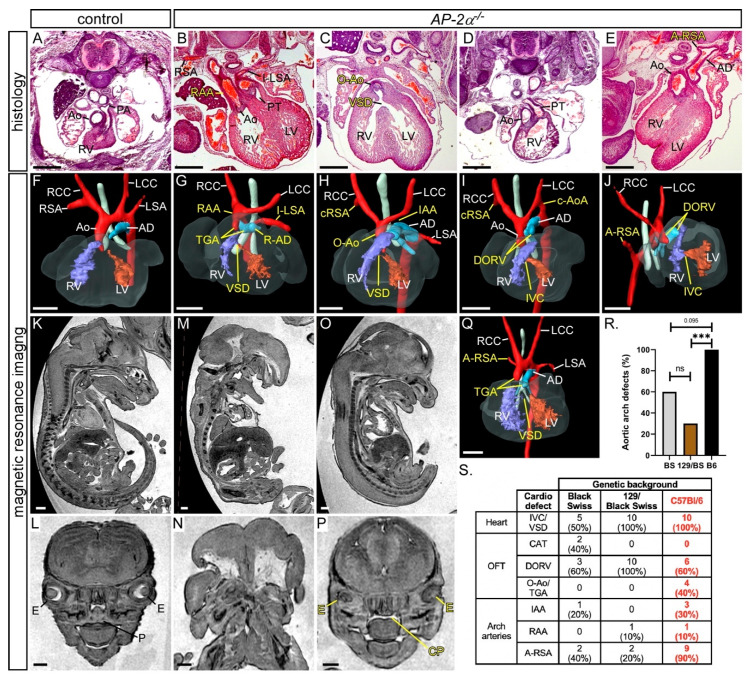
Defects in *AP-2α*^−/−^ embryos. Images were created from haematoxylin and eosin stained transverse sections (**A**–**E**) and MRI datasets (**F**–**P**) from E15.5 embryos. Control *AP-2α*^+/+^ embryos had normal cardiovascular structures (**A**,**F**), bodies (**K**) and palates (**L**). *AP-2α*^−/−^ embryos show a range of defects including transposition of the great arteries (TGA; **B**,**G**,**Q**), right-sided aortic arch (RAA) and arterial duct (R-AD), with an isolated left subclavian artery (I-LSA; **B**,**G**), interruption of the aortic arch (IAA; **H**), over-riding aorta (O-Ao; **C**,**H**), and cervical origin of the right subclavian artery (cRSA; **H**,**I**), cervical origin of the aortic arch (c-AoA; **I**) and double-outlet right ventricle (DORV; **D**,**E**,**I**,**J**), and retro-oesophageal right subclavian artery (A-RSA; **E**,**J**,**Q**). All outflow tract defects were associated with a ventricular septal defect (VSD) or an interventricular communication (IVC; **C**,**G**–**J**,**Q**). *AP-2α*^−/−^ embryos usually also present with severe ventral body wall (**M**) and craniofacial defects (**M**,**N**). One *AP-2α*^−/−^ embryo presented with a mild external phenotype (**O**) and minor craniofacial defects consisting of abnormal eyes (E) and cleft palate (CP; **P**), although this was accompanied by a severe cardiovascular abnormality (**Q**). (**R**,**S**) Effect of genetic background on the *AP-2α*-null cardiovascular phenotype. Data for Black Swiss (BS) and 129/Black Swiss (129/BS) genetic backgrounds reproduced from [24]; C57Bl/6J (B6) data from this study (red font). There is a significant increase in the number of *AP-2α*^−/−^ aortic arch artery defects on the B6 background. *** *p* < 0.005, Fisher’s exact test (**R**). Abbreviations: AD, arterial duct; Ao, aorta; LCC, left common carotid; LSA, left subclavian artery; LV, left ventricle; OFT, outflow tract; P, palate; PT, pulmonary trunk; RCC, right common carotid; RSA, right subclavian artery; RV, right ventricle. Scale, 500 μm.

In this study we wanted to identify the tissue in which AP-2α is required to control normal cardiovascular development. Firstly, however, we analysed the developing cardiovascular system in embryos deficient for *AP-2α* (*AP-2α*^−/−^) on an enriched C57Bl/6J genetic background using histology, magnetic resonance imaging (MRI) and high-resolution episcopic microscopy (HREM). *AP-2α*^+/−^ mice, which have an IRES-*lacZ* sequence inserted to disrupt the dimerization and DNA binding domains of the AP-2α protein [24], were intercrossed and embryos collected at E15.5 for analysis. This revealed that all *AP-2α*^−/−^ embryos (*n* = 10) analysed had a ventricular septal defect (VSD) or an interventricular communication with an OFT defect (10/10), as shown in Figure 1 and Table 1, with six embryos showing a double outlet right ventricle, as shown in Figure 1D,E,I,J, two showing an over-riding aorta, as shown in Figure 1C,H, and two exhibiting transposition of the great arteries (TGA). One embryo with TGA also had an aberrant right subclavian artery (A-RSA), a right-sided aortic arch and an isolated left subclavian artery, as shown in Figure 1B,G. Although TGA is a hallmark of heterotaxy [48,49], we did not observe any other left–right patterning defects in *AP-2α*^−/−^ embryos. Other aortic arch artery defects were also observed, including interrupted aortic arch (*n* = 3/10), as shown in Figure 1H, cervical origin of the aortic arch (*n* = 2/10), as shown in Figure 1I, and A-RSA (*n* = 3/10 retro-oesophageal and *n* = 6/10 with a cervical origin), as shown in Figure 1H–J. All *AP-2α*^−/−^ embryos, except one, also presented with the previously reported craniofacial and ventral body wall defects [22,23]. One embryo, with a closed ventral body wall, had less severe craniofacial abnormalities, presenting only with a cleft palate and abnormal eyes. This embryo displayed a VSD, TGA and A-RSA, as shown in Figure 1O,P,Q. This suggests that the cardiovascular defects seen in *AP-2α*^−/−^ embryos are not secondary to the craniofacial and ventral body wall abnormalities. In comparison to the published data [22,24], the mice used in this study presented with a significantly increased prevalence of aortic arch artery defects, as shown in Figure 1R,S. This, therefore, demonstrates that *AP-2α*^−/−^ embryos on an enriched C57Bl/6J genetic background have fully penetrant cardiovascular defects affecting the aortic arch arteries, as well as the OFT.

**Figure 2:**
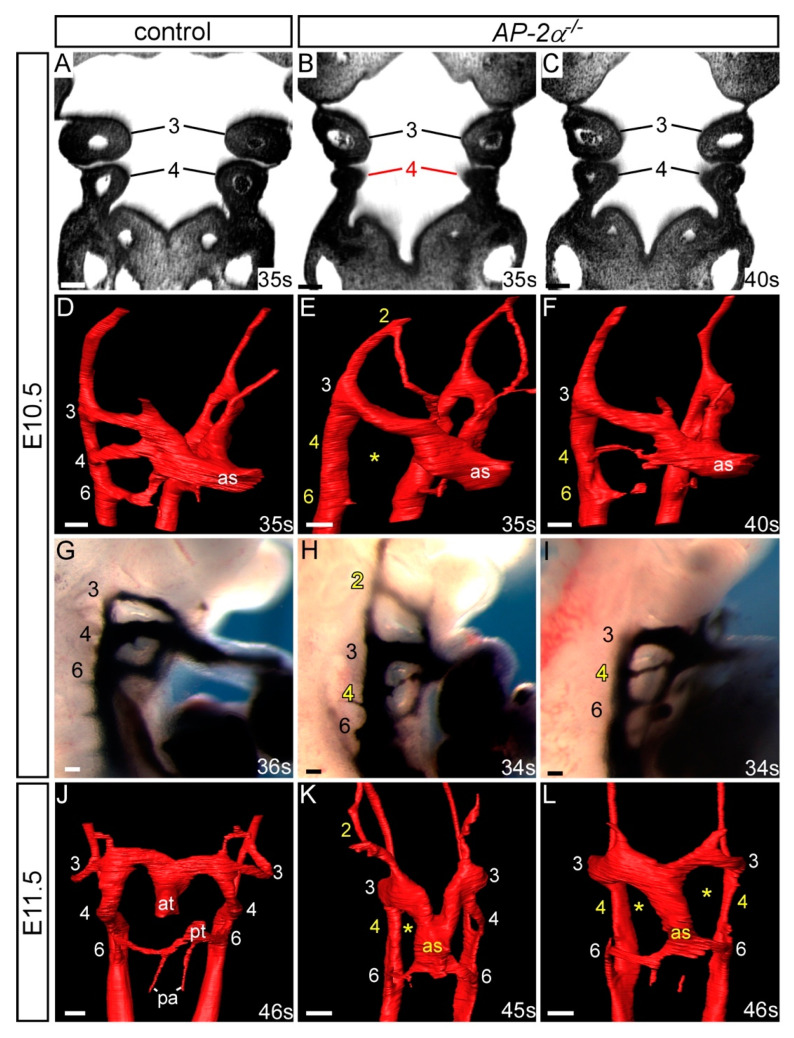
Pharyngeal arch artery patterning defects in *AP-2α*^−/−^ embryos. Images were created from 3-D reconstructions of HREM datasets (**A**–**F**, **J**–**L**) and from ink injections (**G**–**I**). (**A**,**D**) Control *AP-2α*^+/+^ embryos display normal development of the pharyngeal arches (**A**) and PAA (**D**) at E10.5. In *AP-2α*^−/−^, the embryos, the caudal pharyngeal arch can appear hypoplastic (**B**) or normal (**C**), and the PAAs are frequently abnormal showing persistence of the second and absence of the fourth (**E**) or a hypoplastic fourth PAA (**F**). The sixth PAA formation was observed to be delayed in some embryos (**E**,**F**). (**G**–**I**) Ink injection shows that the third, fourth and sixth PAAs are symmetrical and patent to ink in control embryos (**G**). In *AP-2α*^−/−^ embryos the second PAA was patent (**H**) and the fourth hypoplastic (**H**,**I**) or absent (not shown). (**J**–**L**) By E11.5 the outflow tract is septated in control embryos (**J**). In *AP-2α*^−/−^ embryos, the septation of the outflow tract is delayed, and the second PAA is persistent and the fourth PAA is unilaterally (**K**) or bilaterally (**L**) absent. Pharyngeal arches (**A**–**C**) and PAAs (**D**–**L**) are numbered. Somite numbers (s) are given. Abbreviations: at, aortic trunk; as, aortic sac; pa, pulmonary artery; pt, pulmonary trunk. Scale, 100 μm.

As the aortic arch arteries were found to be 100% affected in *AP-2α*^−/−^ embryos on a C57Bl/6J genetic background we next examined whether the PAAs forming during E10.5-E11.5 developed abnormally using HREM (E10.5 to E11.5; 35–47 somites; *n* = 9) and intracardial ink injection (E10.5; 34–39 somites; *n* = 10). By HREM, four *AP-2α*^−/−^ embryos examined at E10.5-E11.0 (35–43 somites), prior to remodelling of the third and fourth PAAs and the OFT, the fourth pharyngeal arch was observed to be smaller than in wild-type embryos, as shown in Figure 2B,C. The second PAA was abnormally persistent in two embryos, the fourth PAAs bilaterally absent or hypoplastic in all four embryos, and the sixth PAAs not yet formed in two embryos, as shown in Figure 2E,F and Table 2. By ink injection at E10.5, control embryos (34–42 somites; *n* = 7) had a symmetrical third, fourth and sixth PAAs that were patent to ink, and the first and second PAAs had regressed, as shown in Figure 2G. In *AP-2α*^−/−^ embryos, 8/10 had a fourth PAA defect where this vessel was either unilaterally (*n* = 3/10) or bilaterally (*n* = 5/10) not patent to ink or hypoplastic in appearance, as shown in Figure 2H,I and Table 2. Moreover, 4/10 embryos also presented with a unilateral abnormally persisting second PAA patent to ink, and delayed formation of the sixth PAAs, as shown in Figure 2H,I and Table 2. At the E11.5 stage in wild-type embryos, the OFT was septated and the right sixth PAA had begun to regress, although the third and fourth PAAs remained symmetrical, as shown in Figure 2J. Of the five E11.5 *AP-2α*^−/−^ embryos examined (45–47 somites), the second PAA was unilaterally persistent in four embryos and the fourth PAAs were either absent or hypoplastic in four embryos, as shown in Figure 2K,L and Table 2. The majority of the unilateral second PAA defects in *AP-2α*^−/−^ embryos at E10.5-E11.5 affected the right second PAA (in 8/9 cases) whereas the fourth PAAs were affected bilaterally in 10/19 cases, with the right fourth PAA affected unilaterally in 4/19, and the left in 2/19 cases. In all *AP-2α*^−/−^ embryos examined by HREM at E11.5 the OFT remained unseptated and the sixth PAAs were bilaterally present, as shown in Figure 2K,L.

**Figure 3:**
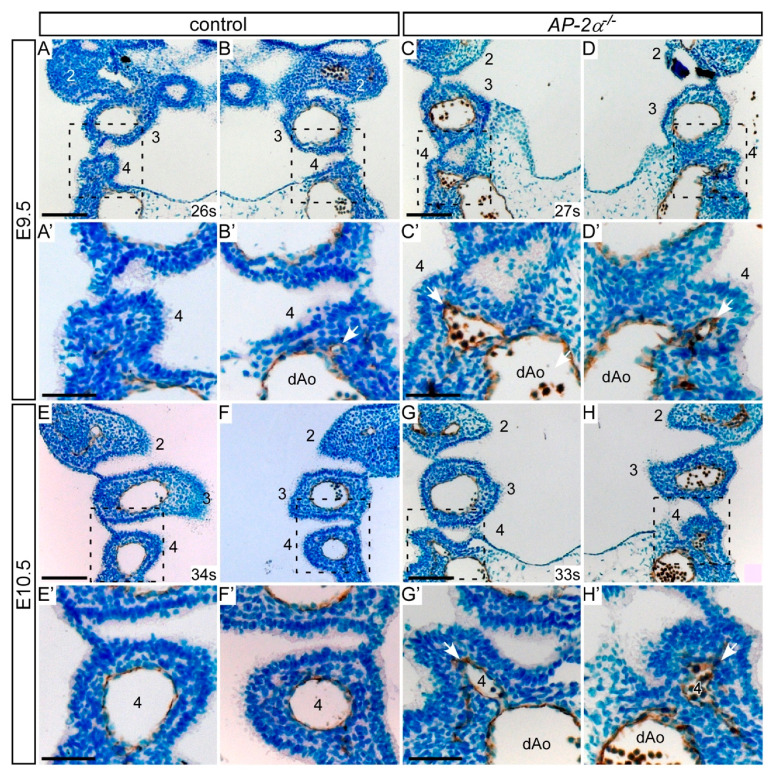
Pharyngeal arch artery development in *AP-2α*^−/−^ embryos. Coronal sections were immunostained with anti-PECAM1 antibody to label endothelial cells of the PAA. (**A**–**D**) During the E9.5 stage the fourth pharyngeal arch is forming but the fourth PAA itself is only beginning to appear in the control and *AP-2α*^−/−^ embryos. PECAM1 positive endothelial cells can be seen budding from the dorsal aorta (dAo; *arrows*). (**E**,**F**) In control embryos at E10.5 the fourth PAAs have formed, have a diameter similar to the third PAAs, and are lined with PECAM1 positive endothelial cells. (**G**,**H**) In *AP-2α*^−/−^ embryos at E10.5 the third PAAs have formed normally but the fourth PAAs are much smaller and appear more irregular in shape (*arrows*). Each PAA is numbered, and somite numbers (s) are given. Scales: (**A**–**G**), 100 μm; (**A’**–**G’**), 50 μm.

To investigate the endothelial cells lining the developing fourth PAA, we immunostained embryo sections with anti-PECAM-1 antibody to label endothelial cells. Embryos were collected at the stages when the fourth PAAa are beginning to form (late E9.5) and have formed (mid E10.5), and five embryos for each genotype (somite range 26–34) were examined. During E9.5, the fourth pharyngeal arch becomes apparent, with PECAM-1 stained endothelial cells of the forming fourth PAA visible close to the dorsal aorta in control (*AP-2α*^+/−^) and *AP-2α*^−/−^ embryos, as shown in Figure 3A–D. In control embryos with 30 to 34 somites, the fourth pharyngeal arch is well defined with the fourth PAA displaying a similar diameter to the third PAAs, surrounded by positively stained PECAM-1 endothelium, as shown in Figure 3E,F. However, in *AP-2α*^−/−^ embryos of an equivalent stage, PECAM-1 positive cells were either clustered and disorganised or had formed a vessel of small diameter, as shown in Figure 3G,H. It therefore appears that the fourth PAA in the *AP-2α*^−/−^ embryos are failing to form correctly.

**Figure 4:**
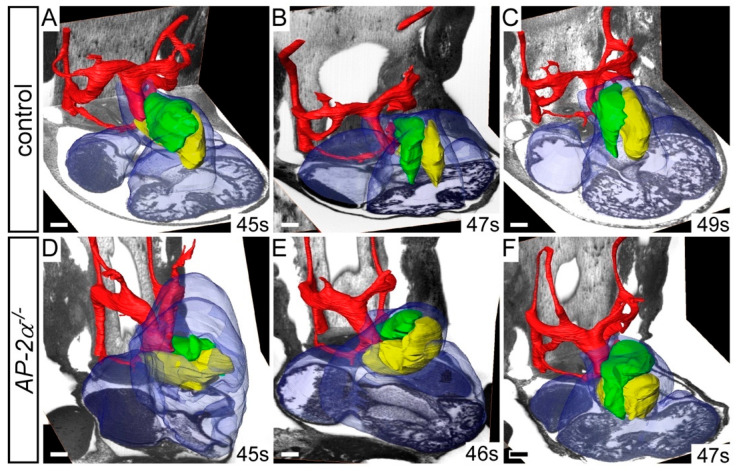
*AP-2α*^−/−^ embryos display aberrant rotation of the outflow tract. Image datasets were acquired by high-resolution episcopic microscopy. (**A**–**F**) Outflow tract rotation during E11.5 (45–49 s). (**A**–**C**) In control embryos, the major outflow tract cushions (parietal in green; septal in yellow) have rotated in an anti-clockwise direction, resulting in the cushions being aligned side by side. (**D**–**F**) In *AP-2α*^−/−^ embryos, the cushions have not rotated correctly resulting in the parietal cushion lying anterior to the septal cushion. Somite (s) counts indicated. Scale bars: 100 μm.

*AP-2α*^−/−^ embryos at E15.5 displayed OFT defects, such as double outlet right ventricle, over-riding aorta and TGA, as shown in Figure 1. We therefore examined the HREM datasets of *AP-2α*^−/−^ embryos at E11.5 to see if malrotation of the OFT cushions could explain these phenotypes. During mid-E11.5, the major septal and parietal cushions are positioned side-by-side, as shown in Figure 4A–C. In *AP-2α*^−/−^ embryos, however, the OFT cushions were mal-positioned with the parietal cushion more anterior to the septal cushion, as shown in Figure 4D–F, reminiscent of the orientation of the cushions at the early E11.5 stage [16].

These results demonstrate that the cardiovascular phenotype of *AP-2α*^−/−^ embryos on an enriched C57Bl/6J genetic background, as assessed by histology, imaging, ink injections and immunostaining, is caused primarily by the fourth PAA not forming correctly, and aberrant OFT alignment with the ventricles.

### 3.2. Cell Fates in AP-2α^−/−^ Embryos

**Figure 5:**
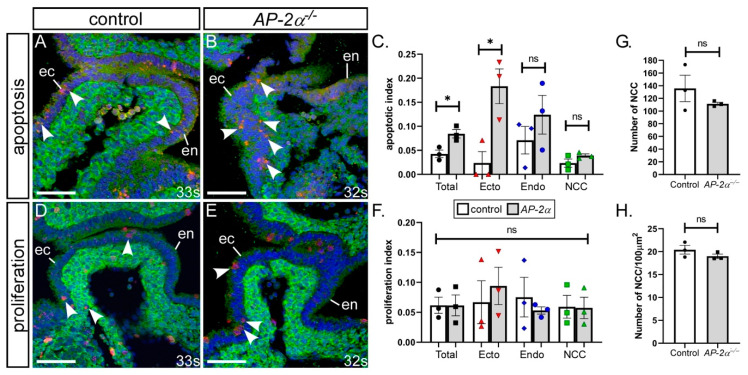
Increased apoptosis in the pharyngeal ectoderm of *AP-2α*^−/−^ embryos. Sections from E10.5 control (*AP-2α*^+/−^; **A**,**D**) and *AP-2α*^−/−^ (**B**,**E**) embryos were immunostained with cleaved anti-caspase3 (*red*; **A**,**B**), anti-phosphohistone H3 (*red*; **D**,**E**) and anti-GFP (*green*; **A**,**B**,**D**,**E**) antibodies to detect apoptosis and proliferating cells within the fourth pharyngeal arch. The number of apoptotic (**C**) and proliferating (**F**) cells were counted and expressed as an index. (**G**,**H**), Neural crest cells (NCC) within the fourth pharyngeal arches of control and *AP-2α*^−/−^ embryos were counted and expressed as the total number (**G**) and the number per 100μm^2^ (**H**). Abbreviations: ec/ecto, ectoderm; en/endo, endoderm. * *p* < 0.05 (two-tailed unpaired *t*-test). Somite (s) counts indicated. Scale, 20 μm.

NCC are important for PAA remodelling [35]. We therefore investigated whether the NCC migrating into the pharyngeal arches were affected in *AP-2α*^−/−^ embryos compared to the controls using *Wnt1Cre* and *eYFP* transgenic mice to lineage trace the NCC and maintain a high percentage of C57Bl/6J genetic background. Immunostaining was performed for NCC using an anti-GFP antibody, and cellular apoptosis and proliferation assessed. Embryos were collected at early E10.5 (somite range 32–34), a stage when the fourth PAAs have formed (*n* = 3 of each genotype). Firstly, apoptosis was assessed in all the cells within the fourth pharyngeal arch by immunostaining for cleaved caspase-3 activity and a significant two-fold increase in the number of apoptotic cells was identified throughout the fourth pharyngeal arch in *AP-2α*^−/−^ embryos compared with control (*p* = 0.023), as shown in Figure 5A–C. When NCC, endoderm and ectoderm pharyngeal arch tissue were analysed separately, we identified a significant 7.7-fold increase in the number of apoptotic cells in the pharyngeal ectoderm compared to control ectoderm (*p =* 0.021), as shown in Figure 5C. The levels of apoptosis in the endoderm and NCC were unchanged compared to the controls. For proliferation, the sections were immunostained for phosphohistone H3 activity. The levels of proliferation within the fourth pharyngeal arches of the control and *AP-2α*^−/−^ embryos were not significantly different, either in total or when each pharyngeal arch tissue was analysed separately, as shown in Figure 5D–F.

As cell death and proliferation within NCC was unaffected we next investigated whether the number and density of NCC present in the pharyngeal arches was altered in *AP-2α*^−/−^ embryos using lineage traced NCC (*n* = 3 of each genotype at E10.5). We found no statistically significant difference in the number or density of NCC within the fourth pharyngeal arches between control and *AP-2α*^−/−^ embryos, as shown in Figure 5G,H.

NCC number and density within the pharyngeal arches were, therefore, not affected in *AP-2α*^−/−^ embryos, nor was cellular proliferation. Increased levels of apoptosis, however, were observed in the pharyngeal ectoderm of *AP-2α*^−/−^ embryos at a critical time of fourth PAA development. This indicates that the cardiovascular defects observed may be derived from increased apoptosis within the pharyngeal ectoderm that consequently affects normal development.

### 3.3. Conditional Deletion of AP-2α from the Pharyngeal Surface Ectoderm

**Figure 6:**
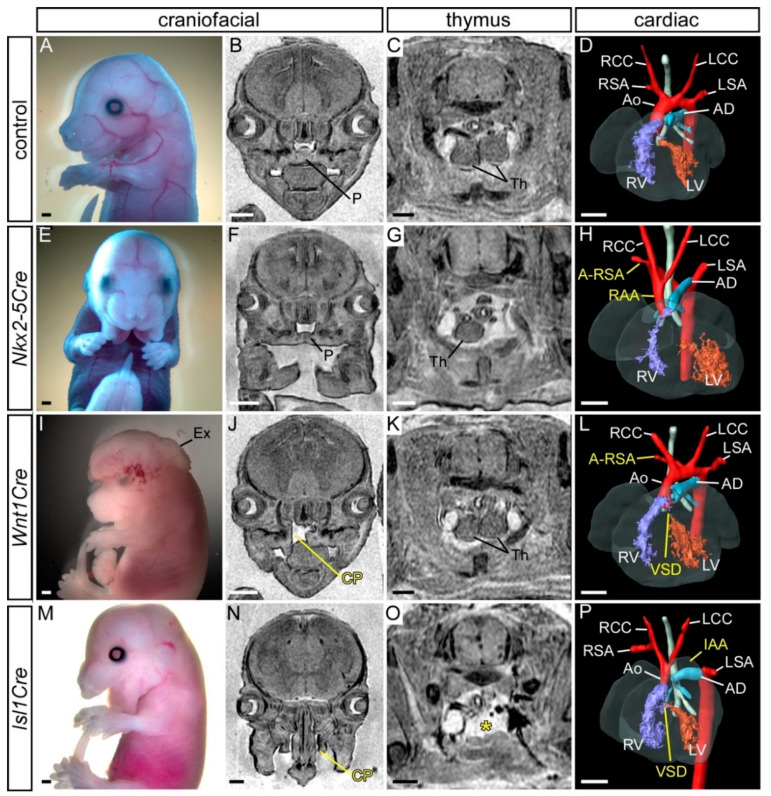
Defects following conditional deletion of *AP-2α*. (**A**–**D**) Control (*AP-2α^+/flox^*) embryo with normal craniofacial appearance (**A**,**B**), thymus (**C**), heart and aortic arch arteries (**D**). (**E**–**H**) Mutant embryo with *AP-2α* conditionally deleted using *Nkx2-5Cre* showing craniofacial (**E**,**F**), thymus (**G**) and a right-sided aortic arch (RAA) and an aberrant right subclavian artery (A-RSA; **H**). (**I**–**L**) Mutant embryo with *AP-2α* conditionally deleted using *Wnt1Cre* showing exencephaly (**I**), cleft palate (CP; **J**), normal thymus (**K**), A-RSA and a ventricular septal defect (VSD; **L**). (**M**–**P**) Mutant embryo with *AP-2α* conditionally deleted using *Isl1Cre* showing craniofacial (**M**,**N**), thymus absent from its normal position (asterisk; **O**), an interrupted aortic arch (IAA) and VSD (**P**). Abbreviations: AD, arterial duct; Ao, aorta; LCC, left common carotid; LSA, left subclavian artery; LV, left ventricle; P, palate; RCC, right common carotid; RSA, right subclavian artery; RV, right ventricle. Scale, 500 μm.

Previous work has demonstrated that, although *AP-2α* is expressed in NCC, conditional deletion using neural crest-specific *Cre* recombinase transgenic mice, *Wnt1Cre* and *P0-Cre*, in conjunction with the *AP-2α^flox^* allele, does not result in cardiovascular defects [36]. As apoptosis was significantly increased in the pharyngeal ectoderm, we next explored the possibility that *AP-2α* expression in this tissue may be critical for cardiovascular development, as this is the only other domain in which AP-2α is expressed during PAA formation, as shown in Figure S1A,B,K. All control embryos analysed with an *AP-2α^flox/+^;Cre* genotype were normal, as shown in Figure 6A–D.

*Foxg1Cre* is expressed in the pharyngeal ectoderm, endoderm and mesoderm from E8.5, but not in NCC, as shown in Figure S1C,D [37,50]. Mutant E15.5 embryos conditionally lacking *AP-2α* from the pharyngeal ectoderm (*AP-2α^flox/−^;Foxg1Cre*) presented with a clefting of the face, and compared to control embryos, the thymus was not observed in its normal position above the heart, or only one lobe was seen in this position (*n* = 7/9), as shown in Figure S2 and Table 3. No cardiovascular defects, however, were observed, as shown in Table 1. This therefore suggests that conditionally deleting *AP-2α* from the pharyngeal surface ectoderm, in combination with endoderm and mesoderm, at least with *Foxg1Cre*, does not give rise to cardiovascular defects. Due to the complex expression pattern of *Foxg1Cre* in the head [37], and to maintain our focus on the cardiovascular system, we did not assess the impact on craniofacial defects further. As this result was unexpected, we employed a second *Cre* transgenic line, *Nkx2-5Cre*, that has been described to be active in the pharyngeal surface ectoderm (and pharyngeal mesoderm and endoderm) from E8.0, but is also not expressed in NCC, as shown in Figure S1E,F [38,50]. Mutant *AP-2α^flox/−^;Nkx2-5Cre* embryos at E15.5 (*n* = 14) presented with a craniofacial defect consisting of a midline cleft of the mandible, as shown in Figure 6E,F and Table 3. The thymus was affected in 5/14 embryos, being either bilaterally or unilaterally absent from the normal position or reduced in size and split asymmetrically, as shown in Figure 6G and Table 3. One *AP-2α^flox/−^;Nkx2-5Cre* embryo presented with a right-sided aortic arch forming a vascular ring that looped around the back of the trachea and joined the descending aorta (therefore interrupted on the left), as shown in Figure 6H and Table 1, and one embryo showed cervical origin of the aortic arch.

The selective deletion of *AP-2α* from the pharyngeal ectoderm using either *Foxg1Cre* or *Nkx2-5Cre* transgenic mice did not, therefore, recapitulate the cardiovascular malformations seen in the global *AP-2α*-null embryos.

### 3.4. Conditional Deletion of AP-2α from the Neural Crest

The possibility that genetic background may have influenced the penetrance of a cardiovascular phenotype in the previously published work raised the question that perhaps the role of *AP-2α* in the neural crest should be re-examined on the inbred C57Bl/6J background. To do this, mice carrying the *Wnt1Cre* transgene were used to conditionally delete *AP-2α* from all NCC [39]. *AP-2α^flox/−^;Wnt1Cre* embryos were examined for cardiovascular defects at E15.5 (*n* = 19). One embryo presented with exencephaly, as shown in Figure 6I, five had a cleft palate, as shown in Figure 6J, and the thymus was unaffected, as shown in Figure 6K and Table 3. Two embryos had cardiovascular defects: one had a perimembranous VSD with A-RSA, as shown in Figure 6L and Table 1, and one had a vascular ring, as described above. We therefore identified a very low incidence (10%) of cardiovascular abnormalities in embryos with a neural crest cell-specific deletion of *AP-2α* on an enriched C57Bl/6J genetic background.

### 3.5. Simultaneous Deletion of AP-2α from the Surface Ectoderm and the Neural Crest

AP-2α is expressed in both NCCs and the pharyngeal surface ectoderm, as shown in Figure S1A,B,K. As deletion from either tissue individually did not recapitulate the level of cardiovascular defects seen in the global mutants, we next investigated the possibility that *AP-2α* is required in both tissues simultaneously for correct cardiovascular development. We therefore created mutant mice that would delete *AP-2α* from the pharyngeal ectoderm and NCC simultaneously. Male mice heterozygous for *AP-2α* and transgenic for both *Wnt1Cre* and *Foxg1Cre* were crossed to *AP-2α^flox/flox^* females, and embryos collected at E15.5 for analysis. A craniofacial defect was observed in all embryos of the genotype *AP-2α^flox/−^; Wnt1Cre;Foxg1Cre* (*n* = 6) similar to that seen in Figure S2. In addition, exencephaly was observed (2/6) as well as thymus defects, as shown in Table 3. By histology, no cardiovascular defects were observed, although an A-RSA was identified in one *AP-2α^flox/−^;Wnt1Cre* embryo from this cohort, as shown in Table 1. From these data, it appears that the simultaneous deletion of *AP-2α* from the NCC and surface ectoderm using *Wnt1Cre* and *Foxg1Cre* is unable to cause cardiovascular defects in mutant embryos. We therefore employed *Nkx2-5Cre* in conjunction with *Wnt1Cre* to delete *AP-2α* from the surface ectoderm and NCC together. All *AP-2α^flox/−^;Wnt1Cre;Nkx2-5Cre* mutant embryos (*n* = 9) showed a facial cleft similar to that shown in Figure 6E,F, and one had a thymus defect, as shown in Table 3. One *AP-2α^flox/−^;Wnt1Cre;Nkx2-5Cre* mutant embryo had a perimembranous VSD and cervical origin of the right subclavian artery, as shown in Table 1.

In an attempt to delete *AP-2α* from the pharyngeal ectoderm at a slightly earlier time than with *Nkx2-5Cre* and *Foxg1Cre*, we used *Isl1Cre* mice [40]. Isl1 is expressed in the SHF as early as E7.0 [51] and throughout the developing pharyngeal arches in the pharyngeal endoderm, ectoderm and mesoderm, and in NCC, as shown in Figure S1I,J [43,52,53]. The *Isl1Cre* transgenic line will therefore delete *AP-2α* from the surface ectoderm and NCC component of its expression domain. Mutant *AP-2α^flox/−^;Isl1Cre* embryos (*n* = 11) at E15.5 were analysed and showed craniofacial and thymus defects, as shown in Figure 6M–O and Table 3, and four mutants presented with a cardiovascular defect, displaying perimembranous VSD with either interrupted aortic arch or A-RSA, and a hypoplastic aorta, as shown in Figure 6P and Table 1.

Taken together, the conditional, and simultaneous deletion, of *AP-2α* from the pharyngeal ectoderm and/or NCC, somewhat unexpectedly, did not fully recapitulate the cardiovascular defects seen in the global *AP-2α* null embryos. These results indicate that the simultaneous conditional deletion of *AP-2α* from the NCC and the surface ectoderm does not result in highly penetrant cardiovascular malformations, at least not with the *Cre* lines employed here.

### 3.6. Early Embryonic Recombination of the AP-2α^flox^ Allele

Because of the unexpected lack of a penetrant cardiovascular phenotype with any of the *Cre* lines used in this study, we decided to closely analyse the cardiovascular phenotype produced by a global deletion of the *AP-2α^flox^* allele and compare this to the phenotype seen in the constitutive *AP-2α* deficient embryos.

**Figure 7:**
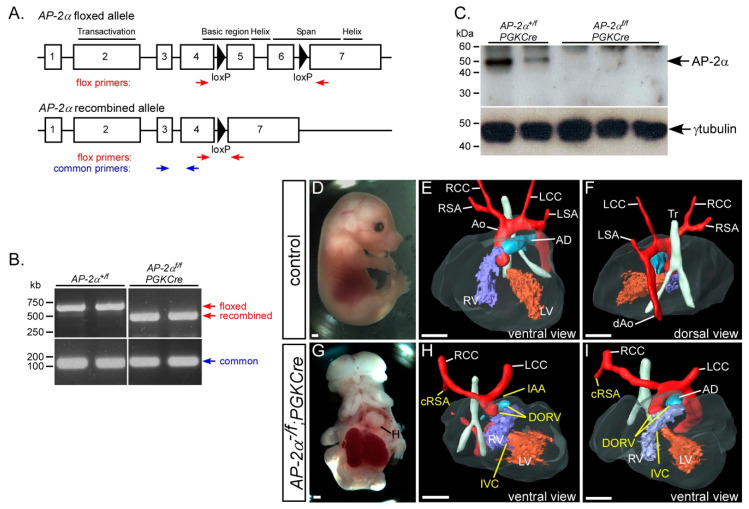
Defects following global conditional deletion of *AP-2α*. (**A**) Cartoon of the *AP-2α^flox^* allele [36] with the functional domains, *loxP* sites (black triangles) and genotyping primers indicated. (**B**) RT-PCR analysis demonstrates that the *AP-2α^flox^* allele is fully recombined with *PGKCre*. (**C**) No AP-2α protein is produced following the PGKCre-mediated recombination of the *AP-2α^flox^* allele. γ-tubulin antibody was used as a loading control. (**D**–**F**) Control (*AP-2α^+/flox^;PGKCre*) embryos were externally normal (**D**) and had a normal heart, outflow tract and aortic arch arteries (**E**,**F**). (**G**–**I**) Conditional deletion of *AP-2α* using *PGKCre* (*AP-2α^−/flox^;PGKCre*) produced embryos with the typical *AP-2α*-null external phenotype (**G**) and cardiovascular defects, including interruption of the aortic arch (IAA), cervical origin of the right subclavian artery (cRSA) and double-outlet right ventricle (DORV) with interventricular communication (IVC; **H**,**I**). Abbreviations: AD, arterial duct; Ao, aorta; dAo, dorsal aorta; LCC, left common carotid; LSA, left subclavian artery; LV, left ventricle; H, heart; RCC, right common carotid; RSA, right subclavian artery; RV, right ventricle; Tr, trachea. Scale, 500 μm.

To achieve the recombination of the *AP-2α^flox^* allele at an early embryonic stage, we used *PGKCre* transgenic mice, which have been reported to express Cre recombinase in all tissues from E3.5 [41]. To confirm that the *AP-2α^flox^* allele, as shown in Figure 7A, was being fully recombined we performed RT-PCR on cDNA made from E10.5 embryo RNA. This demonstrated that the *AP-2α^flox^* allele was correctly recombined in the presence of *PGKCre*, as shown in Figure 7B. We also showed that no truncated AP-2α protein was made by Western blotting, as shown in Figure 7C. These data demonstrated that the *AP-2α^flox^* allele was completely recombined and created a null allele. To investigate the cardiovascular system, stud males of the genotype *AP-2α^+/−^;PGKCre* were created, and crossed to *AP-2α^flox/flox^* females to generate mutant (*AP-2α^flox/−^;PGKCre*) and control (*AP-2α^flox/+^;PGKCre*) embryos at E15.5 for analysis. The external phenotype clearly recapitulated the *AP-2α*^−/−^ external phenotype, as shown in Figure 7G [22,23,24]. All *AP-2α^flox/−^;PGKCre* mutant embryos presented with an aortic arch artery defect and 70% with an OFT defect, which was not significantly different from the fully penetrant OFT defects seen in the global *AP-2α* deficient embryos, as shown in Figure 7H, I and Table 1.

These data indicate that cardiovascular defects equalling the prevalence seen in *AP-2α*^−/−^ embryos can only be recapitulated conditionally with an early recombination of the *AP-2α^flox^* allele throughout the embryo in conjunction with the *AP-2α* null allele.

## 4. Discussion

In this study we have defined the cardiovascular phenotype in mice lacking *AP-2α* on a C57Bl/6J genetic background, although we were unable to fully recapitulate the cardiovascular phenotype seen in *AP-2α* deficient embryos using a conditional deletion strategy from the neural crest and/or pharyngeal surface ectoderm. The complete *AP-2α* deficient phenotype, was, however, achieved through very early embryonic recombination of the *AP-2α* conditional allele.

AP-2α is expressed in the NCC and the pharyngeal ectoderm of the pharyngeal arches that house the PAAs that will form and remodel to create the aortic arch arteries that take blood away from the heart [24,33,34]. Conditional deletion of *AP-2α* from the NCC and/or the pharyngeal surface ectoderm, failed to recapitulate the *AP-2α*-null cardiovascular phenotype, although a small percentage displayed an arch artery defect. The conditional deletion of *AP-2α* using *Isl1Cre* did produce the highest number of embryos with a cardiovascular malformation (36%) of all the Cre combinations used. *Isl1Cre* is expressed throughout the SHF including the pharyngeal epithelia [43,52], but it has also been reported to be active in the cardiac neural crest [53]. It could therefore be speculated that the earlier onset of *Isl1Cre* expression [51], combined with activity in the pharyngeal surface ectoderm and NCC, could have sufficiently recombined the *AP-2α^flox^* allele to produce a limited cardiovascular phenotype. The overall timing of the expression of the Cre transgenes used in this study, however, may therefore be too late to successfully fully recombine the *AP-2α^flox^* allele. By employing *PGKCre* mutant mice to conditionally delete *AP-2α* before gastrulation, the full *AP-2α*-deficient phenotype was successfully recapitulated. This is analogous to the *Cited2* knockout mouse, where the full heterogenous cardiovascular and laterality phenotype could only be realized conditionally through deletion at gastrulation [54]. Each Cre line used in this study, however, was able to produce mutant embryos with craniofacial defects, demonstrating that these lines functioned correctly and were able to recombine the *AP-2α^flox^* allele and impact the formation of these structures later in development. *Foxg1Cre*, for example, has a complex expression pattern in the head itself [37], but we have not assessed how this pattern might cause the observed craniofacial defects, nor with the other Cre strains used in this study. Nevertheless, the high incidence of craniofacial and thymus defects in the conditional mutants reflects the efficacy of *AP-2α* deletion resulting from the Cre transgenes and supports our conclusions concerning the lack of defects caused by loss of *AP-2α* in relation to the cardiovascular system. A recent study has demonstrated that biallelic deletion of *AP-2α* and *AP-2β* from NCC results in more severe craniofacial defects than seen in single NCC mutants [55], indicating that functional redundancy occurs between these two genes. It is possible, therefore, that cardiovascular development may also be affected more severely in these mutants, providing an alternative explanation as to why so few *AP-2α^flox/−^;Wnt1Cre* mutant embryos presented with cardiovascular defects here, or not at all in the earlier report [36]. Recently it has been recognized that the *Wnt1Cre* line has ectopic overexpression of Wnt1 in the midbrain, which increases Wnt/ß-catenin signalling, and a new allele has been developed, *Wnt1Cre2*, to overcome this issue [56]. This line, however, has been reported to alter NCC-related phenotypes compared to *Wnt1Cre* [57]. Although we do not predict that ectopic midbrain Wnt1 overexpression using the *Wnt1Cre* allele would affect the cardiovascular system in our study, it is feasible that we may have achieved a different result using the *Wnt1Cre2* allele.

Previous studies have described the cardiovascular defects in *AP-2α*-null embryos and demonstrated a cardiovascular phenotype consisting of a fully penetrant OFT malformation and a partially penetrant arch artery phenotype [24]. Here, using mice on an enriched C57Bl/6J genetic background, we have described the fully penetrant OFT and arch artery defects in *AP-2α*-null embryos, demonstrating that *AP-2α* is critical for the correct development of the cardiovascular system. We did not, however, find any embryos with a common arterial trunk, as previously reported [24], but did identify other OFT defects of over-riding aorta and TGA, with the latter not previously described in *AP-2α*^−/−^ mutant embryos. This difference in the penetrance of arch artery defects, and the range in OFT abnormalities observed, may be attributed to the different genetic backgrounds of the mice used in the various published studies. In our study, all the mice used were calculated to be >90% C57Bl/6J for each line used, whereas the mice in the previous work were on a Black Swiss [22] or a mixed genetic background [24]. The *AP-2α*-null alleles were constructed differently, with the former using a neomycin cassette to replace exon 6, and the latter having an IRES-lacZ cassette inserted into exon 7 of the *AP-2α* gene (also used in this study). Although the presence of a neomycin cassette may influence neighbouring gene expression that could impact any observed phenotype [58], it is more likely that the increased penetrance of arch artery defects observed in our study are due to changes in genetic background that are influencing the presentation of specific phenotypes, as previously shown for *Cited2* and *Gbx2*, among other genes involved in cardiovascular development [15,21].

The PAA defects observed between E10.5 and E11.5 in *AP-2α*-null embryos were predominantly affecting the fourth PAAs, either unilaterally or bilaterally, with the development of this artery appearing to be arrested between E9.5 and E10.5, as shown by PECAM1 staining. Failure of the left fourth PAA to form will result in the interruption of the aortic arch and cervical origin of the aorta, and failure of the right fourth PAA will lead to A-RSA, either retro-oesophageal or with a cervical origin [59]. The destiny of the persistent second PAA is more difficult to ascertain; it may regress at a later time in development or may even contribute to the mature aortic arch topology as a replacement vessel for an absent or aberrantly remodelled one, as is seen in *Pax9*-null embryos [16]. Interestingly, the right fourth PAA appeared to be more susceptible to the deletion of *AP-2α* with 100% of the constitutive and the conditional mutants by the foetal stage displaying a defect affecting the right fourth PAA derived vessel (e.g., retro-oesophageal and cervical origin of the right subclavian artery). Moreover, almost half of the embryos at E10.5–E11.5 had an abnormal right second PAA. Mouse embryos, heterozygous for *Tbx1,* present more frequently with A-RSA than interruption of the aortic arch [16,60], indicating that a right-sided bias does occur in other mouse models of cardiovascular malformation. Although it has been described that the *Tbx1*^+/−^ fourth PAA phenotype does partially recover during development [16,17,61], this does not seem to be the case in *AP-2α*-deficient mice, as the incidence of right fourth PAA derived defects is fully penetrant.

*AP-2α*-null embryos display severe neural tube and ventral body wall closure defects (primary thoracoabdominoschisis) [22,23] that may occasionally affect the pericardium [24]. Neural tube defects, such as spina bifida, and foetal thoracoabdominal anomalies, such as gastroschisis and omphalocele, occur concomitantly with CHD [62,63,64]. It is therefore possible that the *AP-2α*-null cardiovascular phenotype occurs concomitantly, or even secondarily, to the body wall closure defects. This is, however, unlikely, as we identified one *AP-2α*-null embryo with an intact ventral body wall and only a mild craniofacial defect that presented with complex OFT and arch artery defects. Although all *AP-2α*-null embryos present with cardiovascular defects incompatible with post-natal life, they are sufficient to support a circulation in the foetus. Following birth, the closure of the duct would result in the rapid demise of the neonate and death within 48 h. However, given the severity of the other defects, it is highly likely that these would independently result in a more immediate post-natal death. For example, the severe craniofacial, body wall, cranial nerve and skeletal defects would prevent feeding and breathing [22,23]. By E10.5, *AP-2α* is no longer expressed in NCCs, but continues to be expressed within the pharyngeal ectoderm [24]. NCC migration is unaffected in *AP-2α*-null embryos [23] and we did not see any defects in NCC apoptosis, proliferation, number or density within the pharyngeal arches, but did observe a significant increase in the number of apoptotic cells in the fourth pharyngeal arch ectoderm of *AP-2α*-null embryos. Increased apoptosis has also been observed in the third and fourth pharyngeal arch ectoderm of *Eya*-null embryos [65], and these mice also display complex cardiovascular defects, such as interruption of the aortic arch and A-RSA that are seen in *AP-2α*^−/−^ embryos [66]. Interestingly, a role for apoptosis in other tissue compartments has been described in animal models of cardiovascular developmental defects. Within the pharyngeal mesenchyme of *Tgfb2*-null mouse embryos at E11.5, apoptotic cells have been observed surrounding the PAAs just before they begin to regress [67]. At later stages, aberrant apoptotic cells were located in the fourth PAAs leading to arch artery defects, such as interrupted aortic arch. Aberrant apoptosis within the tunica media of the arteries has also been implicated in the development of arch artery defects in diabetic rats [68]. To see if a second wave of apoptosis is contributing to the *AP-2α*-null cardiovascular phenotype, the examination of embryos at later stages when the PAA are remodelling (i.e., E11.5–E13.5) would need to be undertaken. At the stage of development analysed in our study, it is not possible to identify the apoptotic ectodermal cells beyond that of a monolayer of columnar, polarised epithelial cells, joined by tight and adherens junctions. The pharyngeal surface ectoderm, however, has been implicated as an important signalling centre for PAA morphogenesis [17,69], so it is possible that AP-2α expression in the ectoderm plays a critical role in mammalian PAA development, akin to the role of ectodermal *tfap2a* in controlling zebrafish skeletogenesis and the promotion of pharyngeal ectoderm survival [70]. Moreover, for the process of ventral body wall closure during development, AP-2α is required for signalling from the surface ectoderm to the underlying mesoderm [71]. As NCC are sensitive to external signals as they migrate [72] we speculate that aberrant signals from the affected pharyngeal ectoderm may alter their behaviour and ability to properly orchestrate the process of OFT septation and ventriculo-arterial connections in *AP-2α*^−/−^ embryos, leading to delayed OFT septation, aberrant endocardial cushion rotation and subsequently giving rise to the observed OFT defects. Septation does finally occur in the *AP-2α*-null embryos as no case of common arterial trunk was observed in our mutants. The OFT cushions in the *AP-2α*-null embryos were aberrantly positioned with the parietal cushion more caudal to the septal cushion, an organisation seen at an earlier stage in development in control embryos [16]. The OFT phenotypes observed, therefore, could be attributed to an alteration in the aortic to pulmonary valve axis angle, following an arrest in normal OFT rotation [73,74]. It should be noted, however, that the aberrant positioning of the OFT cushions in *AP-2α*-null embryos may not represent how the observed defects occur. An alternative mechanism for the correct positioning of the aorta and pulmonary trunk over the correct ventricles is through an asymmetrical lengthening of the right ventricular OFT, which causes the pulmonary trunk to be pushed towards its definitive position in front of the aorta by an asymmetric population of SHF cells [75]. In *Lrp2*-null mice, for example, the abnormal migration of this SHF population, along with defective NCC migration, results in common arterial trunk [76]. Further studies will be required to see if the SHF pulmonary push population has been affected in *AP-2α*-null embryos.

## 5. Conclusions

In this study we have shown that the complex cardiovascular defects in embryos deficient in *AP-2α* are fully penetrant on a C57Bl/6J genetic background. Conditional deletion experiments suggest that *AP-2α* has a complex influence on cardiovascular development by being required very early in embryogenesis, having a redundant function in multiple tissue layers, or a combination of both factors.

## Figures and Tables

**Table 1:** Cardiovascular defects observed in *AP-2α* mutant embryos.

Genotype	*n*	Abnormal	VSD	DORV	O-Ao	TGA	IAA +/−A-RSA	cAoA +/−A-RSA	RAA +/−A-SA	A-RSA
*AP-2α* ^−/−^	10	10 (100%)	4	6	2	2 *^a^*	3	2	1	4
*AP-2α^−/f^;Foxg1Cre*	9	0	-	-	-	-	-	-	-	-
*AP-2α^−/f^;Nkx2-5Cre*	14	2 (14%)	0	0	0	0	0	1	1	0
*AP-2α^−/f^;Wnt1Cre*	25 *^b^*	3 (12%)	1	0	0	0	0	0	1	1
*AP-2α^−/f^;* *Foxg1Cre;Wnt1Cre*	6	0	-	-	-	-	-	-	-	-
*AP-2α^−/f^;* *Nkx2-5Cre;Wnt1Cre*	9	1 (11%)	1	0	0	0	0	0	0	1
*AP-2α^−/f^;Isl1Cre*	11	4 (36%) *^c^*	2	1	0	0	2	0	0	1
*AP-2α^−/f^;PGKCre*	10	10 (100%)	1	7 *^d^*	0	0	1	4	1	4

*^a^* One *AP-2α*^−/−^ embryo with TGA also had a right-sided arterial duct. *^b^ AP-2α^−/f^;Wnt1Cre* embryos from *AP-2α* and *Wnt1Cre* breeding (*n* = 19) and complex *Foxg1Cre;Wnt1Cre* breeding (*n* = 6). *^c^* One *AP-2α^−/f^;Isl1Cre* embryo also had a hypoplastic aorta. All OFT defects (i.e., DORV, O-Ao and TGA) were associated with a VSD or an interventricular communication. *^d^* The number of OFT defects in *AP-2α^−/f^;PGKCre* embryos (7/10) is not significantly different to *AP-2α*^−/−^ embryos (10/10; *p* = 0.211, Fisher’s exact test). Abbreviations: A-RSA, anomalous right subclavian artery (i.e., retro-esophageal, cervical or isolated); A-SA; aberrant right or left subclavian (as A-RSA but also includes isolated left subclavian artery); cAoA, cervical aortic arch; DORV, double outlet right ventricle; IAA, interrupted aortic arch; O-Ao, overriding aorta; OFT, outflow tract; RAA, right-sided aortic arch; TGA, transposition of the great arteries; VSD, ventricular septal defect.

**Table 2:** Pharyngeal arch artery defects in *AP-2α*^−/−^ embryos.

Genotype	*n*	PAA	Abnormal	Unilateral Defect	Bilateral Defect	Present	Hypo/Int/Absent	Absent
*AP-2α* ^−/−^	19	2	11 (58%)	10	1	1	0	0
		4	16 (84%)	6	10	-	8	2
		6	11 (58%)	5	6	-	4	2

Embryos were collected and assessed for PAA defects. *AP-2α*^−/−^ mutants were either injected intracardially with ink (E10.5; *n* = 10) or imaged using HREM (E10.5-E11.5; *n* = 9) to visualise the PAA. Data are combined. For each embryo, left and right PAA 1–6 was scored as having a unilateral or bilateral defect, and the bilateral defects categorised as either present, a combination of hypoplastic, interrupted and/or absent (Hypo/Int/Abs), and bilaterally absent. The first and third PAA were unaffected in all embryos examined. All control *AP-2α*^+/−^ embryos (*n* = 9) were normal.

**Table 3:** Extra-cardiovascular defects in conditionally deleted *AP-2α* embryos.

Genotype	Thymus	Craniofacial
*AP-2α^−/f^;Foxg1Cre*	Bilaterally or unilaterally absent from normal position, reduced in size and/or split (7/9)	Upper facial cleft (7/9)
*AP-2α^−/f^;Nkx2-5Cre*	Bilaterally or unilaterally absent from normal position, reduced in size and/or split (5/14)	Midline cleft mandible (14/14)
*AP-2α^−/f^;Wnt1Cre*	Not affected	Cleft palate (5/19); exencephaly (1/19)
*AP-2α^−/f^;* *Foxg1Cre;Wnt1Cre*	Bilaterally or unilaterally absent from normal position (6/6)	Upper facial cleft (6/6); exencephaly (2/6)
*AP-2α^−/f^;* *Nkx2-5Cre;Wnt1Cre*	Small, split (1/9)	Midline cleft mandible (9/9)
*AP-2α^−/f^;Isl1Cre*	Absent from normal position (9/11)	Midline cleft mandible and upper facial cleft (8/11); cleft palate (6/11); exencephaly (1/11)

## Supplementary Materials

The following are available online at https://www.mdpi.com/2308-3425/7/3/27/s1. Figure S1. E9.5 mouse embryos stained to show expression of lacZ from the *AP-2a^lacZ^* allele. Figure S2. Non-cardiac defects following conditional deletion of *AP-2*α using *Foxg1Cre*.
