# Supplementary material for: Early Embryonic Expression of AP-2α Is Critical for Cardiovascular Development
Source: J Cardiovasc Dev Dis. 2020 Jul 23;7(3):27. doi: 10.3390/jcdd7030027 (PMC7570199; doi:10.3390/jcdd7030027)
Supplement: Supplementary file 1 [file jcdd-07-00027-s001.pdf]

Supplementary files

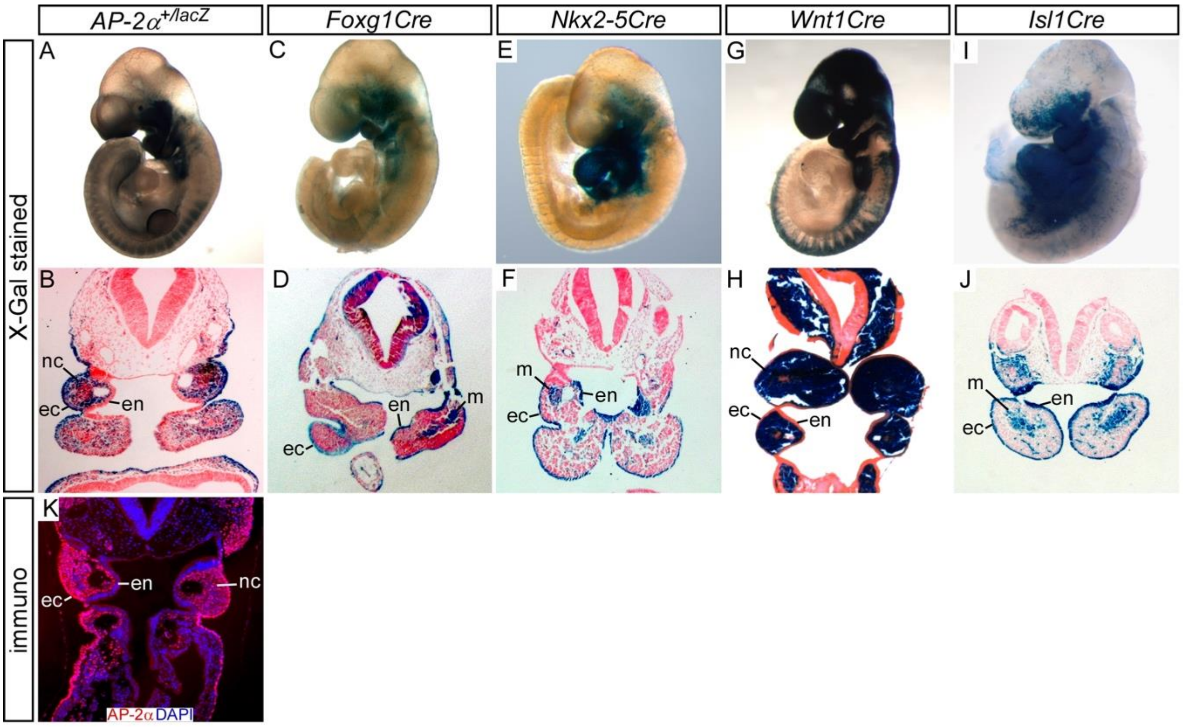

Figure S1. E9.5 mouse embryos stained to show expression of lacZ from the *AP-2α^{lacZ}* allele (**A, B**) and from the *R26R^{lacZ}* allele following Cre-mediated recombination from the alleles used in this study (**C–J**). Whole embryos (**A, C, E, G, I**) and transverse sections counter-stained with eosin (**B, D, F, H, J**) are shown. Note that these Cre transgenes have complex expression patterns outside the pharyngeal region, and we have not assessed how these patterns might cause the observed craniofacial defects. (**K**) Coronal section of a control E9.5 embryo showing AP-2*α* protein expression in the pharyngeal ectoderm and neural crest cells by immuno-staining. Abbreviations: ec, pharyngeal ectoderm; en, pharyngeal endoderm; m, mesoderm; nc, neural crest cells.

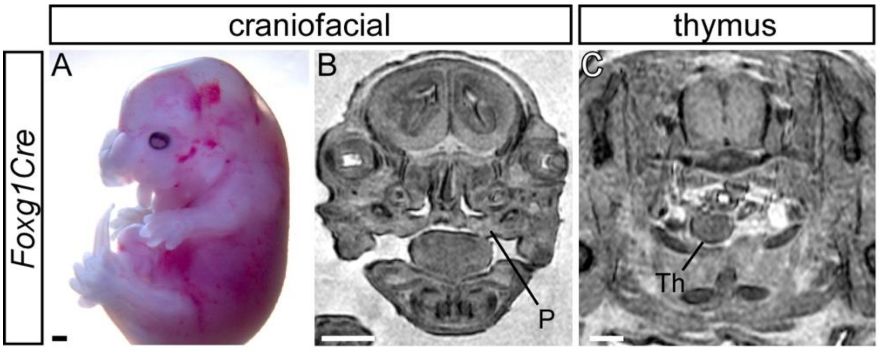

Figure S2. Non-cardiac defects following conditional deletion of *AP-2α* using *Foxg1Cre*. Embryos at E15.5 show craniofacial (**A, B**) and thymus (**C**) defects. Abbreviations: P, palate; Th, thymus. Scale, 500 μm.
